# Graphene Quantum Dots Interfaced with Single Bacterial Spore for Bio-Electromechanical Devices: A Graphene Cytobot

**DOI:** 10.1038/srep09138

**Published:** 2015-03-16

**Authors:** T. S. Sreeprasad, Phong Nguyen, Ahmed Alshogeathri, Luke Hibbeler, Fabian Martinez, Nolan McNeil, Vikas Berry

**Affiliations:** 1 Department of Chemical Engineering, University of Illinois at Chicago, 810 S. Clinton, Chicago, Illinois. 60607, USA

## Abstract

The nanoarchitecture and micromachinery of a cell can be leveraged to fabricate sophisticated cell-driven devices. This requires a coherent strategy to derive cell's mechanistic abilities, microconstruct, and chemical-texture towards such microtechnologies. For example, a microorganism's hydrophobic membrane encapsulating hygroscopic constituents allows it to sustainably withhold a high aquatic pressure. Further, it provides a rich surface chemistry available for nano-interfacing and a strong mechanical response to humidity. Here we demonstrate a route to incorporate a complex cellular structure into microelectromechanics by interfacing compatible graphene quantum dots (GQDs) with a highly responsive single spore microstructure. A sensitive and reproducible electron-tunneling width modulation of 1.63 nm within a network of GQDs chemically-secured on a spore was achieved *via* sporal hydraulics with a driving force of 299.75 Torrs (21.7% water at GQD junctions). The electron-transport activation energy and the Coulomb blockade threshold for the GQD network were 35 meV and 31 meV, respectively; while the inter-GQD capacitance increased by 1.12 folds at maximum hydraulic force. This is the first example of nano/bio interfacing with spores and will lead to the evolution of next-generation bio-derived microarchitectures, probes for cellular/biochemical processes, biomicrorobotic-mechanisms, and membranes for micromechanical actuation.

An endospore is robust, resilient, and highly responsive to water vapor^1^,^2^,^3^ due to their exceptionally hygroscopic biomolecular construct. Such a structure is challenging to realize *via* lithographical/physical routes. Bacillus spore exhibits superior water-responsive energy-density^1^, where structurally, spore can be considered as a stretchable microscale membrane-enclosure (~4–5 μm) of water (~60% in protoplast^4^) with a peptidoglycan protective layer consisting of crosslinked N-acetylmuramic acid (NAM) and N-acetylglucosamine (NAG). It consists of a core with dipicolinic acid, which reduces the water content in the core to 30%, a cortex with ~60% water, and the peptidoglycan membrane that allows exchange^3^ of 97% water with a diffusion time scale of less than 1 min (*via* cortex)^2^.

Recently, accessible chemistry on the surface of microorganisms such as bacteria^5^,^6^, viruses^7^,^8^, and yeast^9^ has been employed for directed nano-assembly with 0D, 1D and 2D nanomaterial^10^,^11^,^12^,^13^,^14^,^15^. However, in such nano/bio architectures it is essential to leverage the biophysical phenomena (such as cell hydrodynamics, single-cell biochemical transport, or cellular homeostasis) in microorganisms to derive bio-actuated functionality^1^. For example, the quantum-mechanical effects (electron-tunneling, optical-blinking^16^, molecular-mechanics^17^,^18^ or sensing^19^,^20^) can be integrated with species' biochemical potential, mechanics, and thermal cycles. GQDs have cell compatible density, flexible anchorage and pertinent electronic properties for interfacing with microorganisms. In this work, we leverage the NAM architecture on spore-wall to assemble a GQD-network on a single spore and employ the controllable trans-membrane hydraulic transport in spore (pseudo-isotonic to hypertonic condition due to high hygroscopicity of protoplasm) to fabricate a highly-responsive electron-tunneling modulating device. This device operates at single spore level and is the first example of graphene-based cytobot, to the best of our knowledge. In comparison to GQDs on polymer, the response to humidity is an order of magnitude faster^20^ (see supporting document).

GQDs – single-atom-thick sheets of sp^2^ hybridized carbon atoms with nanoscale lateral dimensions – exhibit size, shape and edge dependent electrical properties^20^,^21^. Further, it is known that a complex interplay between the delocalized π-electrons (rendering high conductivity), size (quantum confinement effects), edge states (providing either broad or localized electron distribution), and functionalization (sp^3^ state induced scattering, low electron density and electronic states) governs the electrical properties of GQDs^22^,^23^. Consequently, GQD-networks can exhibit electron-tunneling transport with interjunction electromechanics. In this work, a percolating network of poly-L-Lysine functionalized GQDs (pLGQDs) is electrostatically assembled on the wall of Gram-positive *bacillus subtilis* endospore, where the high-energy sporal hydraulics resulting from water transport through its membrane^1^,^2^,^4^, reversibly modulates the electron tunneling characteristics between the GQDs. The graphene structure provides compatible (and low) density to the sporal structure for easy mechanics and flexibility for directly anchoring its distributed functional groups on spore.

To produce GQDs, graphene nanoribbons (GNRs) synthesized via nanotomy were oxidatively cleaved into GQDs via a process established in our lab^20^,^21^. Briefly, a diamond knife was used to cut highly oriented pyrolytic graphite (HOPG) into nanoscale blocks of graphite. These nanoblocks of desired width were exfoliated into GNRs *via* modified Hummers process^24^,^25^. The, GNRs^20^ (50 mg) were oxidatively cleaved into GQDs with edges functionalized with oxygen functionalities (EfGQDs) in highly acidic condition (Conc: H_2_SO_4_, 20 mL) in the presence of KMnO_4_ (200 mg) and NaNO_3_ (2 g). Care was taken to avoid the temperature rise above 10°C while adding of KMnO_4_. The mixture was kept at 50°C under constant stirring for 2 h. The temperature of the system was then raised to 120°C and allowed to react for 12 h. Subsequently, the reaction was arrested using H_2_O_2_. After the oxidation, the sample was sonicated for 3 h. The samples were washed with dilute hydrochloric acid and made to undergo dialysis for 10 days. After dialysis, the dispersion was sonicated for 15 minutes and kept for further processing.

To culture spores, a pellet of vegetative *bacillus subtilis* from a clean stock on agar gel plate was introduced into sterilized 100 ml of nutrient broth solution (0.13 g/ml nutrient broth (OXOID), sterilized at 121°C for 12 min in an Erlenmeyer flask). The cells were cultured at 31.5°C for 36 hr (shake frequency ~ 60 rpm) to ensure spore formation^26^ and were washed via centrifugation with deionized (DI) water three times (6000 rpm for 10 min and re-suspension in fresh DI water). This removes polysaccharide coating on spore-wall. Finally, the aqueous suspension of spores in DI water was contacted with poly-L-lysine coated 300 nm silica-on-silicon substrate (with pre-deposited electrodes) for cell adhesion. The spores grown were ellipse shaped (diameter: 2.0 to 2.5 μm; length: 3 to 5 μm).

The negatively charged EfGQDs (due to the presence of ionized carboxylic acid, epoxy, hydroxyl, carbonyl, phenol, groups) are functionalized with positively charged poly-L-lysine, which has an affinity to NAMmoiety^27^ on spore wall. Poly-L-lysine was added to EfGQD solution, vortexed for 5 minutes and kept undisturbed for 2 hours to produce positively-charged, poly-L-Lysine functionalized GQDs (pLGQDs). To induce pLGQD's self-assembly on spore wall, the silica chip containing immobilized spore was immersed in the pLGQD solution for 12 h at 25 ± 2°C. Later, the chip was washed with copious amount of distilled water and dried under N_2_ flow. Figure 1 illustrates the complete sequence of process steps to produce the biohybrid and the related structural attributes.

The large-area (Fig. S2a) scanning electron microscopic (SEM) image of as-synthesized EfGQDs and the higher magnification (Fig. S2b) transmission electron micrographs (TEM) confirm the cleavage of GNRs into EfGQDs (~150 nm) (Supplementary information Fig. S2). A representative spore or sporating bacterial cell (out of many smaller sizes) that could span between electrodes has a traverse and conjugate diameters of 4–5 μm and 2μm, respectively (Fig. 2a, 2b). The spore bridged between electrodes has a percolating coverage of pLGQDs (Fig. 1a) with high selectivity. Further details of graphene-cytobot fabrication can be found in supplementary information.

Under Raman spectrometer (532 nm wavelength), EfGQDs exhibited three prominent features (Fig. 2c) namely; the D-band (~1354 cm^−1^) attributed to the breathing mode of sp^2^ carbons activated by presence of defects (functionalized edges and basal plane oxidation in GQDs), a strong and sharp G-band (1588 cm^−1^) representing the E^2g^ mode of the C-C stretching vibration in the graphitic lattice at the Γ-point, and a less strong and slightly broad 2D band (2710 cm^−1^) from the second order two phonon process in graphene^28^,^29^,^30^. The Raman spectrum, the absence of further reaction, and the formation of a stable dispersion in water indicates that the formed GQDs are functionalized, predominantly on the edges. Due to the presence of various functional groups such as ether, epoxide, carbonyl, carboxylic, hydroxyl, phenolic, lactone, and quinine etc. on the surface of completely oxidized graphene oxide quantum dots (GOQDs), similar to GO, the extended double bonded lattice structure is disrupted and should exhibit a high intensity D band in the Raman spectrum. The EfGQDs in the present experiment demonstrated (Fig. 2c) a low-intensity D band and low D/G band ratio (~0.24) implying lesser number of oxidized defects. Further, these defects are hypothesized to be at the edges since, (a) basal-plane oxy-groups act as nucleating points for unzipping of GNRs into smaller GQDs and the final EfGQDs did not reduce in size even after prolonged sonication indicating the absence of any oxidized functionalities on the interior lattice, (b) high-temperature water is known to reduce oxy-functionalized sp3-carbons to sp2 carbons, and (c) EfGQDs form a stable suspension in water, as observed. Hence, from the absence of further reaction and the low-intensity D–band observed in the Raman spectrum, it can be deduced that these are EfGQDs and not GOQDs. Bacterial spore did not show any prominent feature, but a slight fluorescence (upward background). The Raman spectra of the hybrid showed a similar background fluorescence enhancement from the bacterium superimposed on the D, G and 2D bands representing the EfGQDs. This further attests to the assembly of EfGQDs on bacterial spore.

As mentioned above, the pLGQD-cytobot construct includes a spore immobilized on silica-on-silicon chip (300 nm SiO_2_) and spanning predeposited electrodes (300/10 nm Au/Cr) separated by 5 μm (Fig. 2a). The relatively high electrical conductivity of the tested devices adduces the assembly of a percolating network of pLGQDs on bacterial spore. In this GQD-network atop spore, the pLGQDs are linked *via* pLGQD-NAM-pLGQD junctions. Therefore, under an electric field (Fig. 3a), the electron transports between pLGQDs through a tunneling barrier (shown later) from the NAM junctions. For chemically induced doping of graphene, via charge transfer, *p*-type doping results in the stiffening of the G-band, whereas n-type doping softens it. The G and 2D bands in pLGQDs (with poly-L-lysine) softened indicating the n-doping of EfGQDs (Fig. 3b and supplementary information Fig. S3). Here, the positively charged molecules induce n-doping in graphenic materials^31^,^32^. We also observed a decrease in I_2D_/I_G_ ratio pointing towards doping and a slight increase in the D-band intensity attributed to the disorder induced by the adsorption of poly-L-lysine. When the pLGQDs gets anchored onto the spores, with the help of negatively charged NAM moieties on the peptidoglycan membrane, we observed partial restoration of stiffening of both G and 2D bands, pointing towards partial p-doping (as expected, Fig. 3b and supplementary information Fig. S3).

The chip-electrodes were connected to probes attached to a Keithley source-meter, and the tunneling current (versus voltage) and its transient response to hydraulic forces (induced by humidity and pressure) were measured (Fig. 4). During the initial conductivity measurements, the currents increased with time, attributed to repositioning of pLGQDs under electric field. The prepared devices were therefore electrically annealed (applied a potential of 10 V for 10 min) prior to the measurements to allow the pLGQDs on the device to reach an equilibrium position. After electrical annealing, the device exhibited consistent, robust and reproducible response. The total conductivity of the device critically depends on electron tunneling, the probability of which is inverse-exponentially proportional to the tunneling distance. Bacterial spores, being highly hygroscopic, are under hydrated state at room humidity. Hence, a reduction in the external water vapor pressure or relative humidity leads to out-diffusion of internal water from the spore and a resultant decrease in the volume and surface area of the spore. The mass transfer rate through the sporal membrane is directly proportional to the driving force:
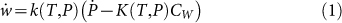
At equilibrium,
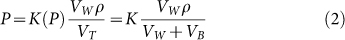
Here, *k(T,P)* and *K(T,P)* are the rate and equilibrium constants, and V_W_, V_B_ and V_T_ are the water, sporal and total volumes in a single spore. Therefore, the total volume of the spore,
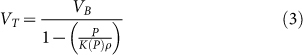
or
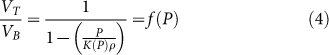
Expanding this expression *via* Taylor series provides: 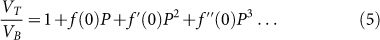
(
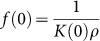
), which is simplified to
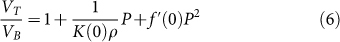
The hygroscopic construct of spore (with dense bio-polyelectrolytes and dipicolinic acid) with ~60% water allows the graphene cytobot-device to sensitively respond to sporal hydraulic forces modulated by external humidity. Here, a fixed voltage was applied to the device, and the transient response of the tunneling current to a change in the local humidity around the device was studied. The local humidity was modulated by passing N_2_ gas (or He, for 30 second intervals), which replaces the humid air around the device. This led to out-diffusion of water through the spore membrane. A sharp increase in conductivity of the device during intervals of N_2_ exposure at three different voltages (Fig. 4a) can probably be attributed to high energy density of spore in response to humidity^1^. Further, the normalized response of the device (current magnitude divided by the average base current) is scaled with voltage (Inset Fig. 5a) attributed to Joule heat induced hydraulic transport through spore wall. A high energy density driven kinetics can be explained for this transient response^1^,^2^, as against diffusion-limited, slow-response kinetics for polyelectrolytes^20^. The control experiments with only GQDs showed high currents but negligible response to humidity; and control experiment without pLGQDs (and only spore) showed low currents at 0.5 V (expected for ionic conductivity). Here, the currents reduced from ~0.5 nA to ~0.1 nA in ~10 seconds. Under vacuum, the current further reduced to the noise level. This is an opposite response direction to that from pLGQD/spore device. Therefore, the spore or the NAM (and NAG) junctions are not (measurably) conductive; however, spore is essential for the functioning of the device. Further, we studied the time of exposure of pLGQD on the deposition density and response, and found no direct correlation. This is attributed to the difference between surface chemistries of different spores. More research is needed to control the spore's surface chemistry accurately to control the deposition density.

Reduction in the volume of the protoplast/cortex/core (due to water loss) and consequent mechanistic shrinking of bacterial spore (due to change in local water-vapor pressure) results in a change in the tunneling distance between the pLGQDs in the hybrid. This can be represented via mass transfer considerations as (see supplementary document):
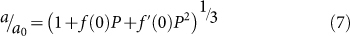
where, *a* and *a_0_* are the average tunneling distances at any point and at zero water-vapor pressure, respectively; and
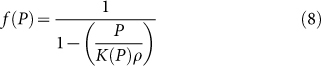
where, *K(P)* is the equilibrium constants, and *ρ* is the spore density. The carrier-transport through the pLGQDs is governed by Fowler Nordheim tunneling (confirmed later)^33^,^34^:
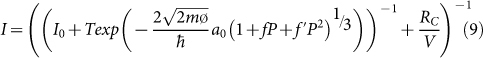
where, *T*, *m*, *ϕ*, *R_C_* are tunneling proportionality constant, mass of an electron, tunneling barrier height, and contact resistance, respectively (see supplementary information).

The hydraulics from the transport of spore-bound water through the wall was also evaluated by exposing the spore-pLGQD device to different levels of vacuum (in vacuum probe-station). The response of the tunneling-currents to the change in external pressure for a spore-pLGQD device and the associated data-fit (Fowler Nordheim fit with regression = 0.995) is shown in Figure 4b. To avoid the mass-transfer limitations, each measurement was recorded after allowing the system to equilibrate (~1–2 min).

Since the electrons transporting out of a pLGQD to the next need to disassociate from pLGQD, the tunneling barrier is assumed to be its work function: 4.85 eV^5^,^18^,^19^,^20^. Our mass-transfer model-fit (supplementary document) shows that the tunneling distance is 0.55 nm for waterless bacteria (from data-fit; Fig. 4b). This value suggests that from 300 Torrs to 0.25 Torrs, the average tunneling-distance reduces by 1.63 nm (from 2.63 nm at room humidity; supplementary information), while the conductivity increases by ~5 folds. The change in electron-tunneling distances with pressure calculated from the model fit is also given in Fig. 4b.

The swelling response of a polymer is related to chemical potential of interaction between the polymer and water^35^,^36^. While the flux of solvent is dependent on the time-dependent chemical potential (μ) as
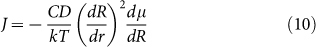
the equilibrium force equation provides:
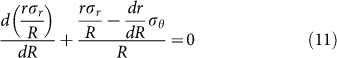
where σ_r_ and σ_θ_ are the swelling pressure applied in the radial and angular directions (cylindrical coordinates). We speculate that the superior response of spore is due to a combined high chemical-potential of spore-constituents/water and a fast diffusion through its membrane (hydrophobic/hydrophilic and selective transport of water). For poly-allyl-hydrochloride fiber with GQDs^20^, the response is >30 seconds: while for spore the response-time is a few seconds (~3 s) (see supplementary information). It is also important to note that while ionic conductivity based humidity sensors are sensitive at higher humidity, and show an increase in conductivity with humidity, the spore/GQD sensor is reverse in response and more sensitive at lower humidity.

To gain further insight into the electron transport mechanism in the spore-pLGQD composite device, four-point probe current–voltage (*I*–*V*) measurements were conducted with eliminated lead resistance (see supplementary information). Under the influence of an external electric field, the charge-carriers tunnel from one pLGQD to the next to produce current. The contact resistance is expected to be smaller than the device resistance and was estimated to be 1201.9 Ω (see supplementary information). Figure 5a shows the IV measured at 80 K, which exhibits a nonlinear response as explicitly shown in the differential curve (dI/dV). This is attributed to 2D Coulomb blockade effects^18^,^37^,^38^, where the transport of charge carriers between neighboring pLGQDs results from a combined and non-synchronous electrostatic charging/discharging mechanism. Furthermore, the increase in currents reduces the residence time of charge carriers and the electrical potential on the upstream electrons. Consequently, the rate of current with voltage increases with the forward bias voltage. This process is modeled *via* the 2D Coulomb-blockade theory^37^ by Likharev: 
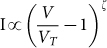
. The Likharev fit provides an estimation of blockade threshold voltage of V_T_ = 31 mV at 80 K and a geometry factor *ζ* = 1.9 (supplementary information Fig. S4). This geometry factor *ζ* = 1.9 is consistent with simulations of electron tunneling in a 2D array of nano particles^39^.

To measure the activation barrier for carrier transport (Fig. 5b) current-voltage (*I–V*, 1–14 mV) measurements were conducted on the pLGQD network at 80, 100, 140, 180 and 200 K. Low voltage range was used to ensure that the electron transport is predominantly tunneling induced due to reduced spilling (supplementary information Fig. S5). The inset of Figure 5b shows the IV-slope (I/V) plot versus 1/T, which fit Arrhenius activation energy behavior: 
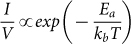
. The activation energy from this fit is 35 meV, which is close to blockade threshold voltage V_T_ = 31 mV (as above). This activation energy is similar to the thermal energy (k_B_T = 25 meV at room temperature), signifying that the mode of electron transport at room temperature is nominally electron tunneling. Here, the Arrhenius equation measures the transport barrier for the lowest tunneling barrier width (under high, fixed vacuum with varied temperature). Since, the Fowler-Nordheim tunneling's dependence on temperature is weak, the barrier measured is transport barrier^34^.

Further, the impedance angle measurements at different frequencies were conducted to deduce the inter-GQD capacitance (C_B_) and water absorption at the NAM junctions between pLGQD assuming a resisto-capacitive pLGQD tunneling junction^40^ (R, C_S_):
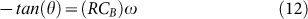
From the slope (intercept = 0) of this equation, the capacitance between pLGQDs was found to increase from 8.54 to 9.61 pF from atmospheric humidity to high vacuum (~3 Torr) (Fig. 6). The small increase (1.12 folds) in capacitance in comparison to larger decrease in the interparticle spacing (4.8 folds from mass-transfer model) can be explained by a decrease of the dielectric constant of the junction ((*ε_w_*/*ε_NAM_*)*f* + (1 − *f*)) under high vacuum. Here, ε_w_ and ε_NAM_ are the dielectric constants of water (80.4) and dry NAM-junction (assumed 5), and *f* is the fraction of water coverage in the tunnel junction (parallel junctions). This provides a water-coverage on spore surface at room humidity as 21.7%, which is consistent with about 50% hydrophilic sites on spore-wall and the 45% humidity (rH) (~22.5% at equilibrium). Since the position and structure of pLGQDs are difficult to decipher, the edges based on contrast gradient are mapped for an FESEM section from Figure 2 (assumed to be pLGQDs boundaries, the top inset of Fig. 6). While there may be several junctions in the pLGQD network, the percolation probability of electron moving from one pLGQD to another will be highest for the junctions with minimum value of
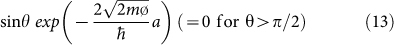
where, θ is the angle between the junction-tangent and the electric field. One such potential path is traced in Figure 6 inset.

In conclusion, we show a unique bio-hydraulic cytobot, where the sharp mechanical actuation of a spore *via* pressure induced water-transport modulates the electron-tunneling width between pLGQDs assembled on its surface. The change in the electron tunneling distance of 1.63 nm due to ~300 Torr change in pressure leads to 5 fold change in the electrical conductivity. The device shows evidence of Coulomb blockade (threshold = 31 mV), and exhibits an activation energy for carrier transport of 35 meV. The device provides an avenue to not only integrate the biological components with 2D nanomaterials; but also to involve bio-structure as an active component in electro-mechanics. Further, spectroscopy confirms the intimate contact between the spore and pLGQDs in this first example of graphene-based ‘biologically-actuating' device. The work provides an avenue for leveraging the unique biomolecular structure of the biological entities to achieve controlled nanoscale architecture and membrane transport for micromechanical actuation for a wide range of applications in microbotics, cellular actuation, single-cell biochemical analysis, cellular homeostasis, specific molecular or ionic detection, and implants for muscle, heart and cancer monitoring.

## Author Contributions

V.B. conceived and directed the research. P.N., T.S.S., A.A., L.H., F.M. and N.M. conducted the humidity experiments; T.S.S. conducted the Raman experiments and structural characterization; P.N. conducted the electrical measurements, and V.B., P.N. and T.S.S. wrote the manuscript. All authors participated in the discussions of the research.

## Supplementary Material

Supplementary Information Supplementary Information

## Figures and Tables

**Figure 1:**
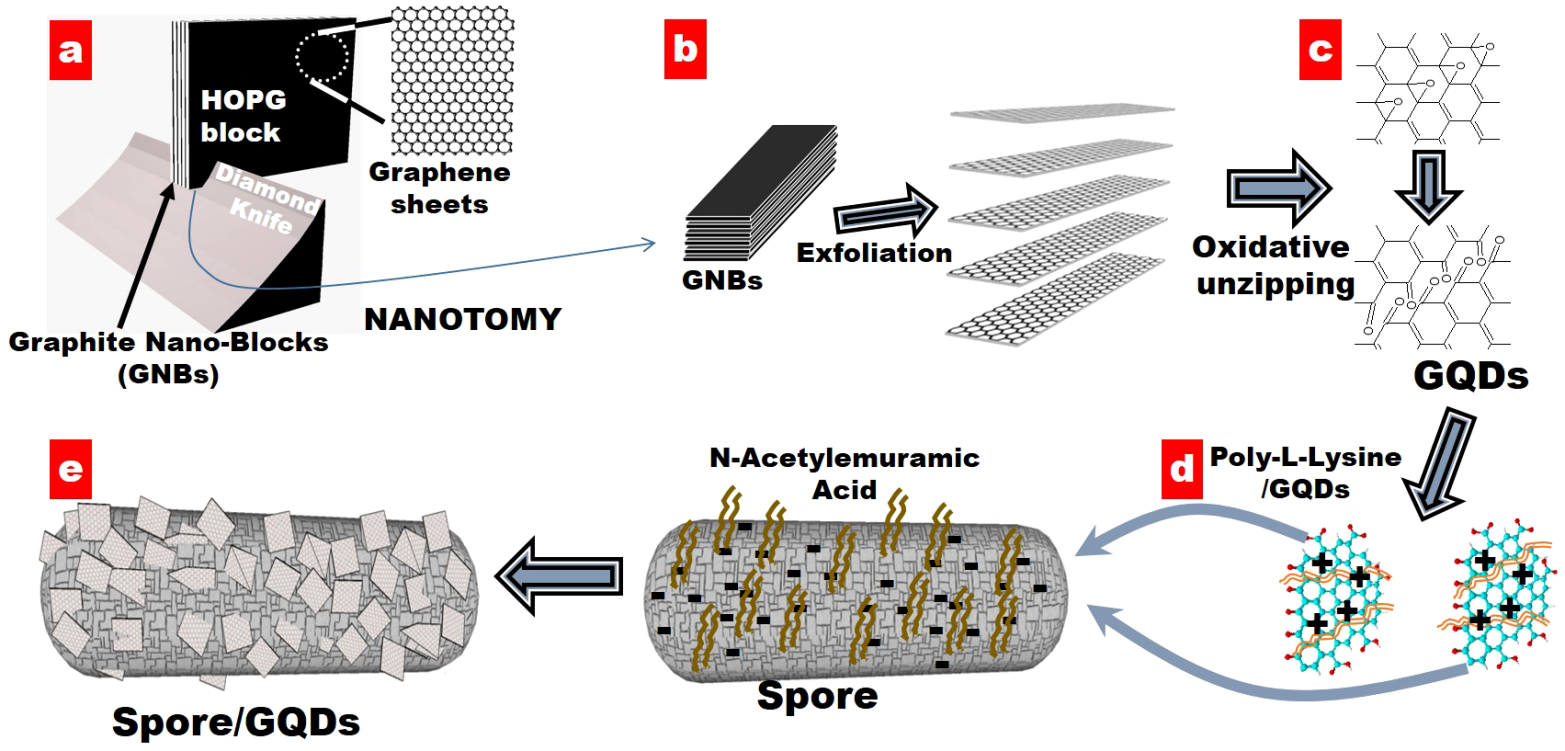
Schematic illustration depicting the formation of GNRs, EfGQDs and pLGQDs, and the immobilization of pLGQDs on spore-wall *via* electrostatic deposition to form the spore-GQD hybrid. (a) A diamond knife cleaves HOPG into GNBs of desired width, (b) GNBs exfoliate into GNRs via oxidation/sonication under mild acidic condition, and (c) as-prepared GNRs unzip into EfGQDs through a second round of vigorous oxidation/sonication with KMnO_4_ and NaNO_3_ in concentrated H_2_SO_4_ (TEM images in supplementary information Fig. S1). (d) EfGQDs are functionalized with poly-L-Lysine to produce pLGQDs, and (e) graphenic cytobots are fabricated by electrostatic assembly: NAM chemistry on the spore wall electrostatically couples with positively charged poly-L-Lysine on pLGQDs resulting in pLGQD/spores bio-hybrid tunneling device.

**Figure 2:**
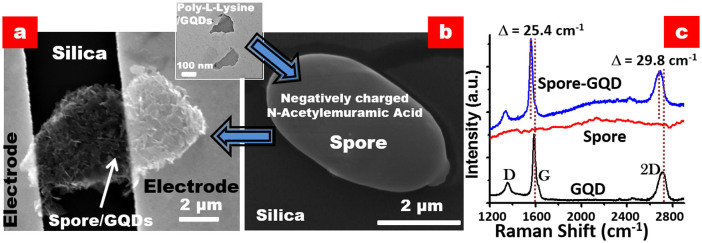
Scanning electron micrographs of (b) a spore on silica and (a) a spore-pLGQD ensemble between electrodes is shown along with a TEM micrograph of pLGQDs. (c) Raman spectra of parent EfGQDs, spore, and the hybrid shows the presence of a luminescent background (from spore) and the characteristic D, G, and 2D bands of pLGQDs establishing the formation of the bio-hybrid.

**Figure 3:**
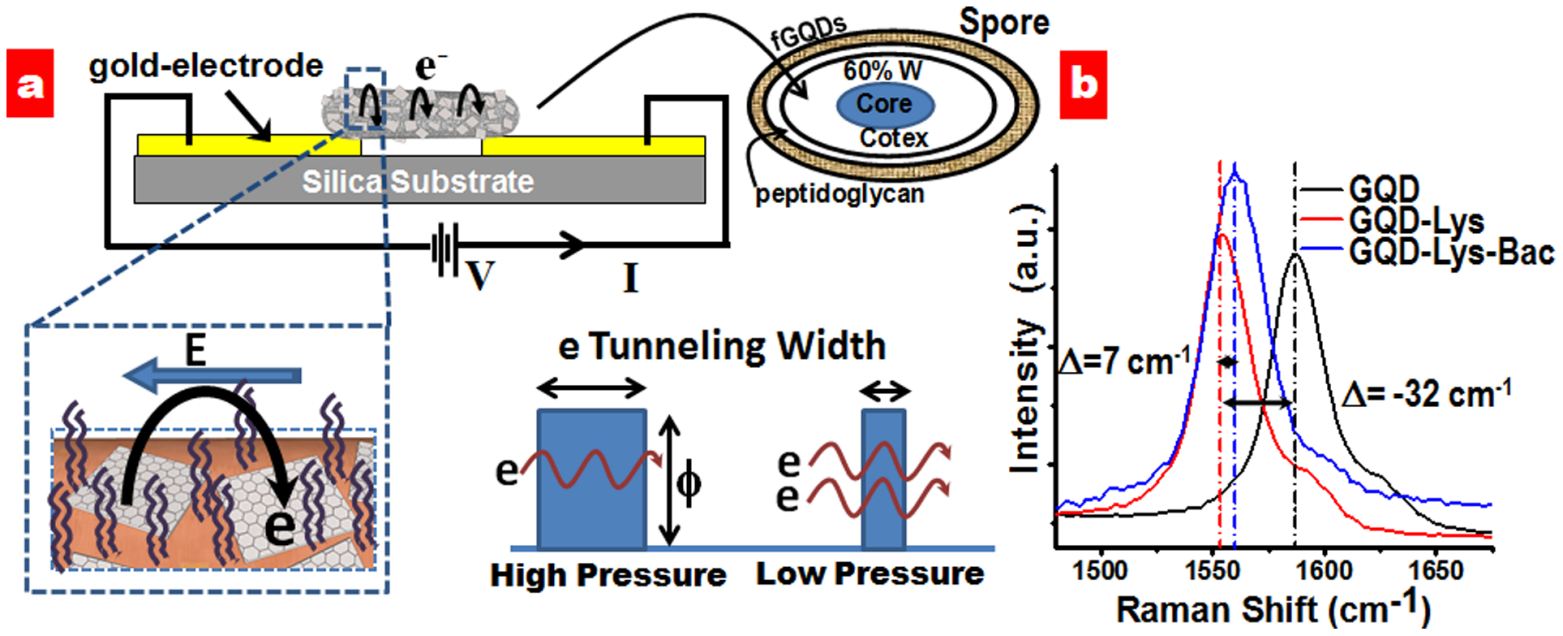
(a) Schematic representation of the cytobot construct and circuitry showing the mechanism for electron-tunneling-modulation in pLGQD-network atop spore. Image is not to scale. (b) n-doping with poly-L-lysine and partially compensated p-doping by spore attachment in pLGQDs results in red-shift and blue-shift in Raman G-band spectra of parent EfGQDs, pLGQDs, and pLGQD-spore.

**Figure 4:**
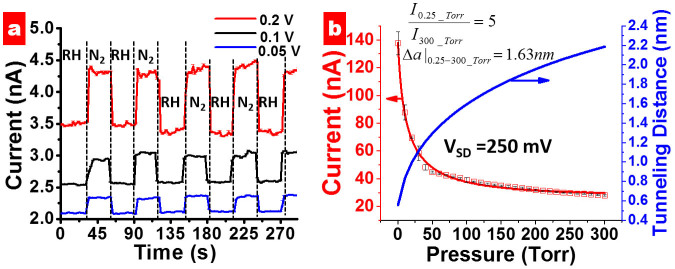
(a) Transient response of the electrical current of the spore-pLGQD hybrid device with change in the local humidity. Multiple cycles of exposure of nitrogen (or helium) gas (30 s exposure time) at different bias voltages (0.05, 0.1 and 0.2 V) points towards the robustness, sensitivity, and fast response of the device. (b) The response of the electrical current in the percolating spore-pLGQD hybrid device at 0.25 V bias with the change in pressure (square points) is shown for a typical device. The model representing the combination of the Fowler-Nordheim electron tunneling and water-transport *via*mass-transfer model fits the data with a regression of 0.996(red curve). The model parameters obtained were used to plot the change in tunneling distances as a function of pressure (blue curve).

**Figure 5:**
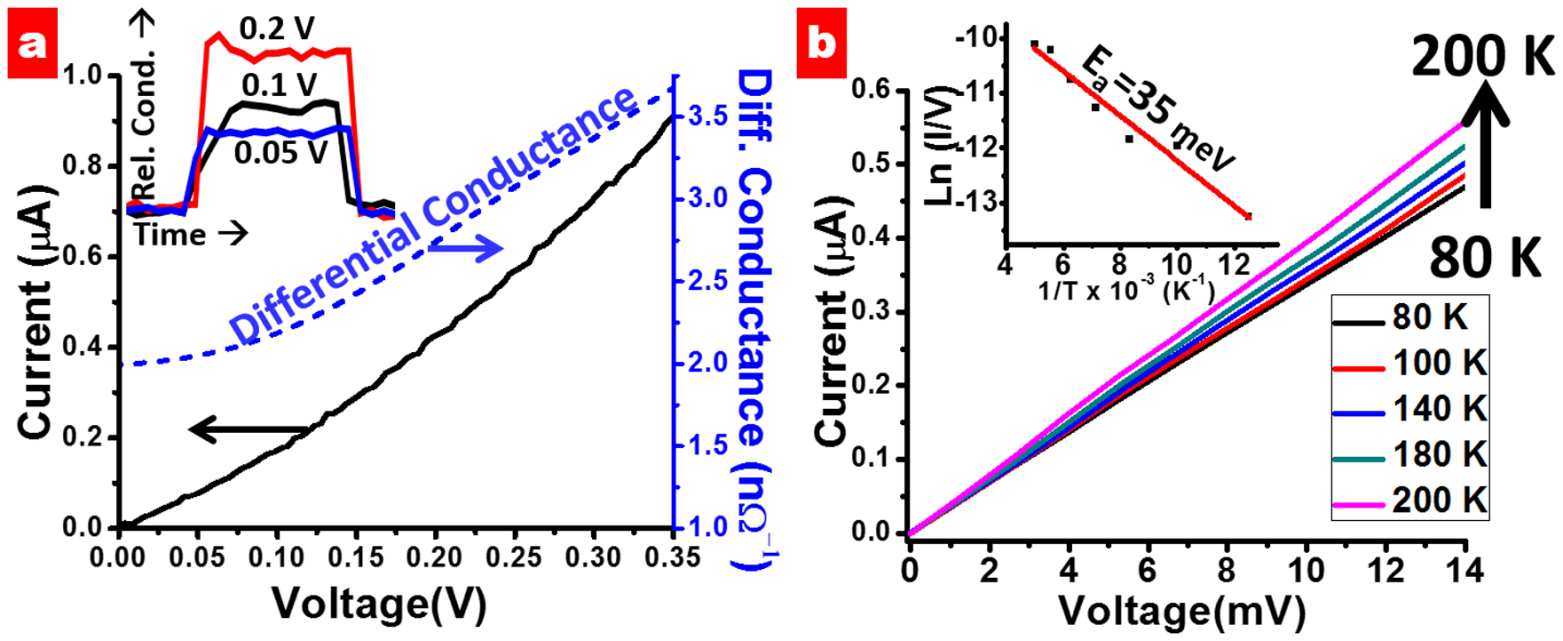
(a) Current–voltage (*I*–*V*) behavior of a spore-pLGQD hybrid device, where the voltage increases from 0 to 0.35 V with a step size of 5 mV (at ~80 K). The differential current (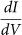) curve represents Coulomb blockade effect in the device. The inset shows the normalized conductivity for one cycle from Fg. 2c at different voltages. (b) The influence of temperature on the electrical currents in the percolating-pLGQD device was studied. The current–voltage (*I*–*V*) curves were obtained at 80 K, 100, 140, 180 and 200 K. (Top-Inset) The decrease in conductivity with decrease in temperature was also plotted with the Arrhenius fit (red curve) to obtain an activation energy of 35 meV (supplementary document).

**Figure 6:**
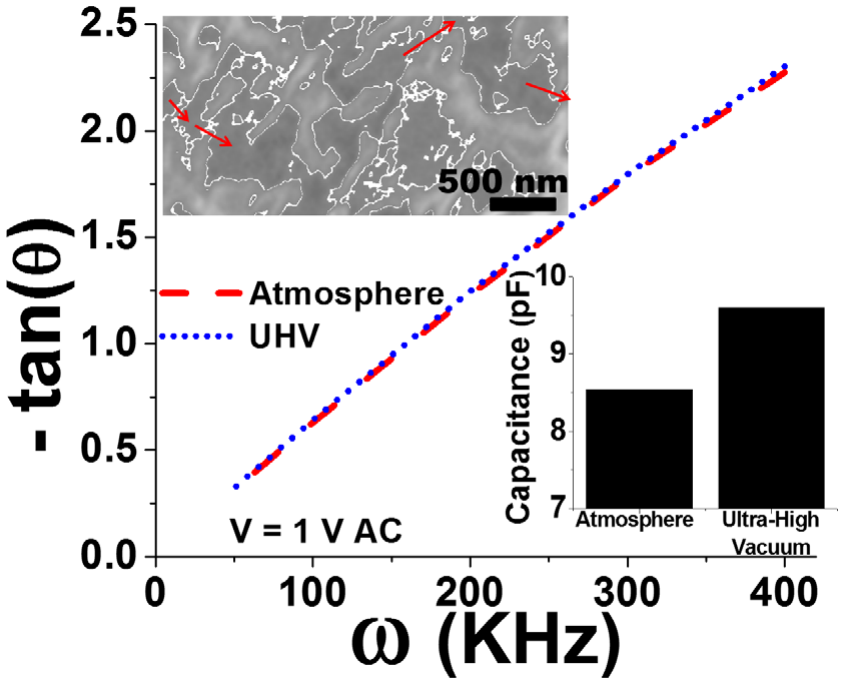
The resisto-capacitive circuit for the pLGQD-network on spore shows a linear relationship between −tan(θ) and frequency at both room humidity and vacuum, as expected (regression = 0.9955). The tunneling capacitance increased from 8.54 to 9.6 pF (bottom inset) upon device exposure to high vacuum from room humidity (45% rH). The top inset shows a section of the FESEM micrograph from Fig. 2 with edges mapped, presumably of pLGQDs on spore. The red arrows show a probable percolation pathway for electron transport.
