# Supplementary material for: Graphene Quantum Dots Interfaced with Single Bacterial Spore for Bio-Electromechanical Devices: A Graphene Cytobot
Source: Sci Rep. 2015 Mar 16;5:9138. doi: 10.1038/srep09138 (PMC4360738; doi:10.1038/srep09138)
Supplement: Supplementary Information [file srep09138-s1.pdf]

Graphene Quantum Dots Interfaced with Single Bacterial Spore for Bio-MEMS Devices: A Graphene Cytobot

T. S. Sreeprasad, Phong Nguyen, Ahmed Alshogeathri, Luke Hibbeler, Fabian Martinez, Nolan McNeil and Vikas Berry*

Department of Chemical Engineering, University of Illinois at Chicago, 810 S. Clinton, Chicago, Illinois, 60607, USA

*vikasb@uic.edu

Table of content:

I. **Graphene cytobot fabrication process:**
1. **Preparation of Graphene Quantum Dots (GQDs)**
2. **Preparation of spores**
3. **Functionalization of 300 nm Si/SiO_2 substrate**
4. **Immobilization of Bacterial spores on poly-Lysine functionalized Si/SiO_2 substrates**
5. **Functionalization of GQDs (fGQds)**
6. **Fabrication of graphene cytobots**

II. **Raman analysis of GQD, fGQD, and Graphene cytobot:**

III. **Electron-Tunneling transport model of Graphene cytobot:**

IV. **Coulomb Blockade model and Four Point Probe I-V measurement of Graphene cytobot:**

V. **Thermal barrier analysis of Graphene cytobot:**

VI. **Capacitance Measurement**

VII. **Response Time**

VIII. **More Devices**

I. Graphene cytobot fabrication process:

1. Preparation of Graphene Quantum Dots (GQDs)

Preparation of GQDs was done via oxidative session of graphene nanoribbons (GNRs) which we reported recently. The GNRs were prepared by the nanotomy process, where a graphite block is cut into graphite nanoblockes (GNBs) of defined dimension and was exfoliated to GNRs.

In this study the exfoliation of GNBs were done in a mild acidic condition. 100 mg of GNBs were added to 5 mL of 6 M H_2SO_4 and was allowed to a balck dispersion. The dispersion was kept in an ice bath and 500 mg of $KMnO_4$ was added. This was stirred for 10 mins and then the temperature was raised to 40 ^oC. The temperature was maintained the same for 2h. After 2h, the temperature was increased to 60 ^oC for 30 mins. Then the heating was removed and the reaction was arrested by the addition of H_2O_2. The product was washed with dil: HCl and was put for dialysis for a week.

The, GNRsA second round of oxidation under harsher condition was done to convert the GNRs to GQDs. GNRs (50 mg) was added to Conc: H_2SO_4, (20 mL) in the presence of $KMnO_4$ (200 mg) and $NaNO_3$ (2g) in an ice-bath. Care was taken to avoid the temperature rise above 10 ^oC while adding of $KMnO_4$. The mixture was then kept at 50^oC under constant stirring for 2 h. The temperature of the system was then raised to 120^oC and allowed to react for 12 h. Subsequently the reaction was arrested using H_2O_2. The sample after the oxidation process was sonicated for 3h. The samples were washed with dilute hydrochloric acid and made to undergo dialysis for 10 days. After dialysis, the dispersion was sonicated for 15 more minutes and kept for further processing.

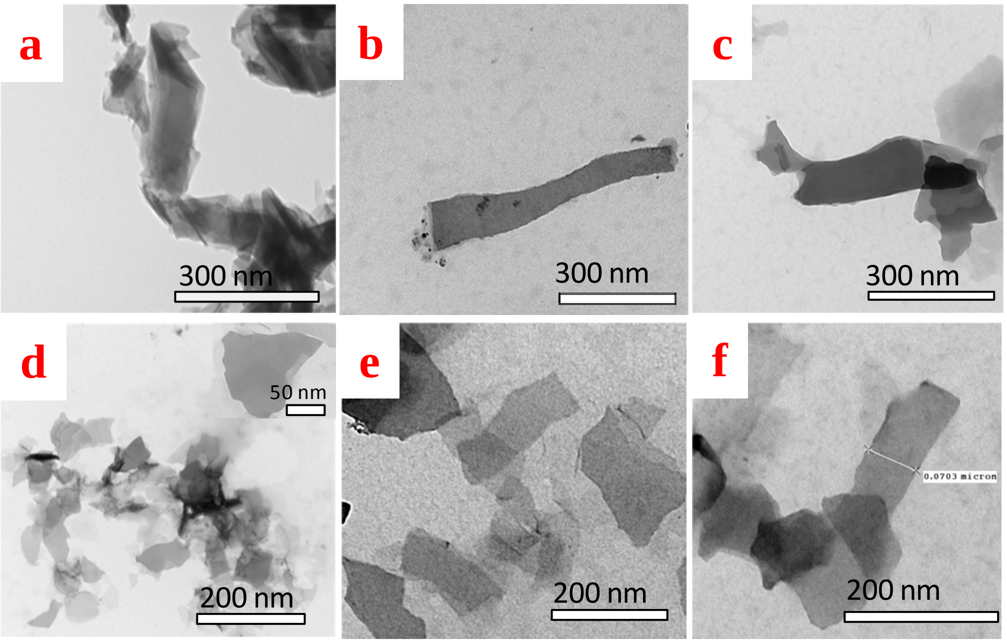

Figure S1. Representative TEM images of size reduction of GNRs and their conversion to GQDs.

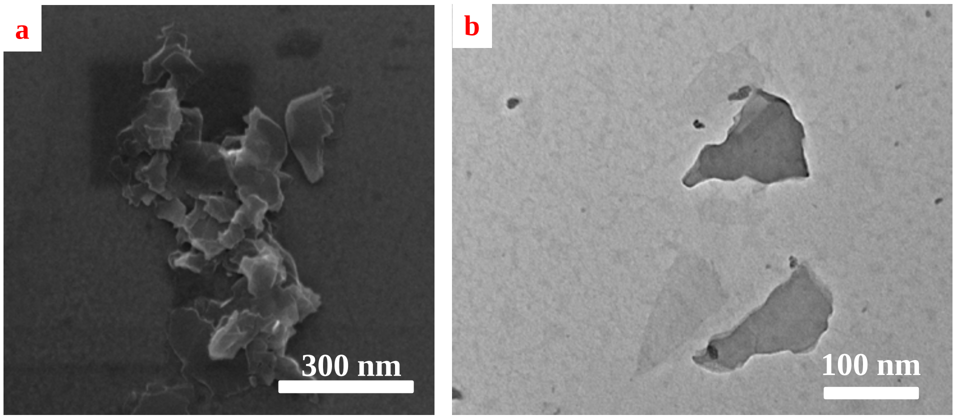

Figure S2. Large area FESEM and magnified TEM image of GQDs

2. Preparation of spores

Bacillus subtilis (from ATCC) stock was smeared on an agar gel plate and grown to ensure there is no cross-contamination of other bacterial species. Using a sterilized culture-transfer rod, a pellet of *bacillus subtilis* cells from this plate was introduced into

100 ml of nutrient broth solution (0.13g/ml nutrient broth (OXOID), sterilized in autoclave at 121 ^{0}C for 12 min) in an Erlenmeyer flask. The flask was sealed with cotton and placed in the incubator to grow the culture at 31.5^{0}C for 36 hr (shake frequency ~ 60 rpm). The grown bacteria were then washed with deionized (DI) water three times by centrifuging the bacterial suspension at 6000 rpm for 10 min and re-suspended in fresh DI water.

3. Functionalization of 300 nm Si/SiO_2 substrate:

To avoid random deposition of functionalized GQDs on the substrate and to aid the attachment of bacterial spores, the substrate was functionalized with poly-Lysine first. The chip was cleaned extensively with water, acetone and isopropyl alcohol (IPA), and was dried under nitrogen flow. The chip was transferred to a plasma chamber and was treated with oxygen plasma(1 Torr, 100 W) for 3 mins. The oxygen plasma treated chip was then immersed in poly-Lysine solution for 2 h facilitate the functionalization.

4. Immobilization of Bacterial spores on poly-Lysine functionalized Si/SiO_2 substrates:

The poly-Lysine functionalized substrates (with/without electrodes) were immersed in the purified spore dispersion and was kept undisturbed for 30 mins. Then the substrates were removed from the dispersion, washed thoroughly with DI water and dried in air.

Each substrate was 1.05 cm X 1.05 cm in area, and included the following number of electrode pairs:

Number of Electrode pairs	Width (or gap between electrodes) (μm)	Electrode length (μm)
10	5	700
4	5	40,000
20	5	50

Different chips (with electrodes) were functionalized with poly-L-Lysine. A series of solutions having different number of bacterial spores were prepared from the cleaned

parent bacterial suspension. The chips were immersed in different vials having different bacterial spore concentrations. Four identical samples were kept for each bacterial concentrations and for each of these samples, the incubation time was varied (5, 15, 30 and 60 min). All these samples were manually examined under the microscope to evaluate the most probable condition to obtain single spore devices. From these sets of experiments, a favorable time (30 min) was obtained where the spores were spaced far apart; but not so less in number that there were no spores bridging the electrode gaps. Further, since we had 34 different (and isolated) electrodes on the chip, the probability to achieve atleast one device with a single spore is very high. In fact, we made about 30 devices at 30 min exposure time and always found a device with a single spore. With 60 min exposure time, the number of spores increases substantially and it becomes challenging to obtain single spore-devices.

5. Functionalization of GQDs (fGQds):

The prepared GQDs were added to a freshly prepared Poly-Lysine solution (2 mg/mL) and was vertexed for 5 mins. Then the sample was kept undisturbed for 2h. For all the experiments, the functionalization was done immediately before the anchoring process on the spores.

6. Fabrication of graphene cytobots

The poly-Lysine functionalized chip was washed again with DI water and was dipped inside a 2 M solution of NaOH. The chip was then washed extensively to make sure that there is no presence of salt in the sample. The chip was then immersed in the fGQDs dispersion for 12 h. After 12h, the chip was removed from the dispersion and was washed repeatedly with DI water to remove the free fGQDs from the devices. The presence of poly-lysine on the chip ensured the removal of free fGQs due to the repulsion between the poly-lysine on GQDs and on the substrate. The devices with single spore bridges between electrodes were identified for electrical characterization

II. Raman analysis of GQD, fGQD, and Graphene cytobot:

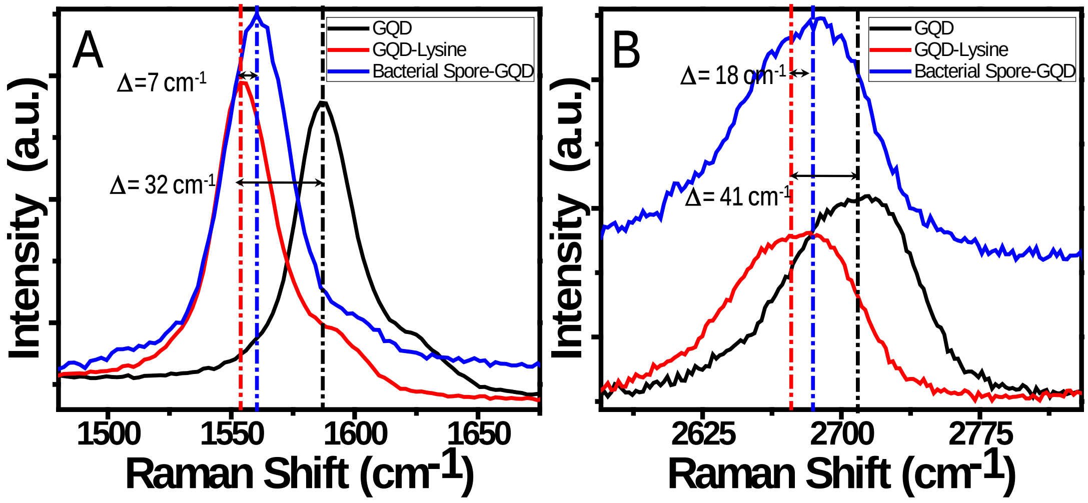

Figure S3. Raman analysis of the bio-hybrid formation: Raman spectra of parent GQDs, fGQDs, bacterial spores and the hybrid showing the enlarged view of (A) the G-band region and (B) the 2D band region. The shifts in the peak position illustrates the functionalization and the corresponding effective doping of GQDs.

The intrinsic physical as well as chemical properties of graphene can be rapidly and nondestructively examined using Raman spectroscopy. The Raman bands (the position, peak shape, as well as intensity) can give intimate details about not only the number of stacked graphene layer, but also the Fermi energy shift(or change in carrier concentration) induced by static electrical field. Hence, Raman spectroscopy can be employed to characterize the nature and extent of doping in graphene. Graphene due to its extreme sensitivity can be easily doped by organic molecules, charged molecular impurities, or by application of electric fields. The Raman G-band of graphene stiffens on both *n* and *p* type, when the doping is induced electrically (electrochemical doping), and consequently G band shifts towards higher wave numbers region. However, when the doping is induces chemically via charge transfer, *p*-type doping results in the stiffening of the G-band, whereas n-type doping softens it. In the present process, we observed a trend similar to the chemical doping, when the samples were analyzed using Raman spectroscopy. Functionalizing GQDs with poly-lysine resulted in the softening

of G and 2D bands indicating the n-doping of GQDs. This is expected as positively charged molecules can induce n-doping in graphenic materials. We also observed a slight increase in the D band, probably due to the disorder induced by the adsorption of poly-lysine. Also we observed a decrease in I_{2D}/I_G ratio again pointing to the doping. When the fGQDs gets anchored onto the bacterial spores, with the help of negatively charged teichoic acid moieties on bacterial spores, we observed stiffening of both G and 2D bands, pointing towards p-doping. The analysis clearly established the formation of the hybrid structure spectroscopically.

III. Electron-Tunneling transport model of Graphene cytobot:

The change tunneling distance between GQD on the bacterial spore is due to the change in the water volume (due to change in local water-vapor pressure) in the spore.

$$ {}^{a}/_{a_0} = (1 + fP + f'(0)P^2)^{1/3} $$

Where, a and a_0 are the average tunneling distances at any point and at zero water-vapor pressure, respectively; and *f* is function of pressure defined below).

The mass transfer rate through the sporal membrane is directly proportional to the driving force: $\dot{w} = k(T,P)(\dot{P} - K(T,P)C_W)$. At equilibrium (assumed instantaneous from electrical results) $P = K(P)\frac{V_W\rho}{V_T} = K\frac{V_W\rho}{V_W+V_B}$. Here, *k(T,P)* and *K(T,P)* are the rate and equilibrium constants, and V_W, V_B and V_T are the water, sporal and total volumes in a single spore. From here, the total volume of the spore comes out to be $V_T = \frac{V_B}{1-\left(\frac{P}{K(P)\rho}\right)}$ or $\frac{V_T}{V_B} = \frac{1}{1-\left(\frac{P}{K(P)\rho}\right)} = f(P)$.

Now we can expand this equation via Taylor series and we get $\frac{V_T}{V_B} = 1 + \acute{f}(0)P + f'(0)P^2 + f''(0)P^3$ ..., here $\acute{f}(0) = \frac{1}{K(0)\rho}$.

This gives $\frac{V_T}{V_B} = 1 + \frac{1}{K(0)\rho}P + f'(0)P^2$ ... The values of the tunneling distance is 0.55 nm.

The carrier-transport through the device is governed by Fowler Nordiem tunneling:

$$ I = \left(\left(I_0 + Texp\left(-\frac{2\sqrt{2m\emptyset}}{\hbar}a_0(1 + fP + f'P^2)^{1/3}\right)\right)^{-1} + \frac{R_C}{V}\right)^{-1} $$

where, *T*, *m*, ϕ , R_C are tunneling proportionality constant, mass of an electron, tunneling barrier height, and contact resistance, respectively (tunneling barrier height, 4.85 eV).

Origin software was used to fit the pressure versus current data. The fitted equation was used:

$$ y = ((c_1 + c_2exp[-2.24 \times c_3((1 + c_4x + c_5x^2)^{0.333})])^{-1} + c_6/0.25) $$

After fitting, the reduce chi-square value was 2.24399 x 10^{-18} and the regression was 0.99596

The values of the parameter are estimated as follows:

$I_o = c_1$	2.67588 x 10^{-8}
$T = c_2$	3.88433 x 10^{-7}
$a_o = c_3(nm)$	0.54667 ~ 0.55
$f(0) = c_4$	0.25293
$f'(0) = c_5$	-1.42332 x 10^{-4}
$RC = c_6$	1201.897

IV. Coulomb Blockade model and Four Point Probe I-V measurement of Graphene cytobot:

Each GQD-Bacterial spore device was studied under 4 probe method to by bass the contact resistance. Likharev fit model is used to fit our experimental data, which provided a blockade threshold voltage (V_T= 31 meV for GQD-Bacterial spore device at 80 K) and geometry factor ζ of 1.9. The numerical simulations of 2D array of nano particles provides geometry factor ζ of 2, which is similar to our experimental values.

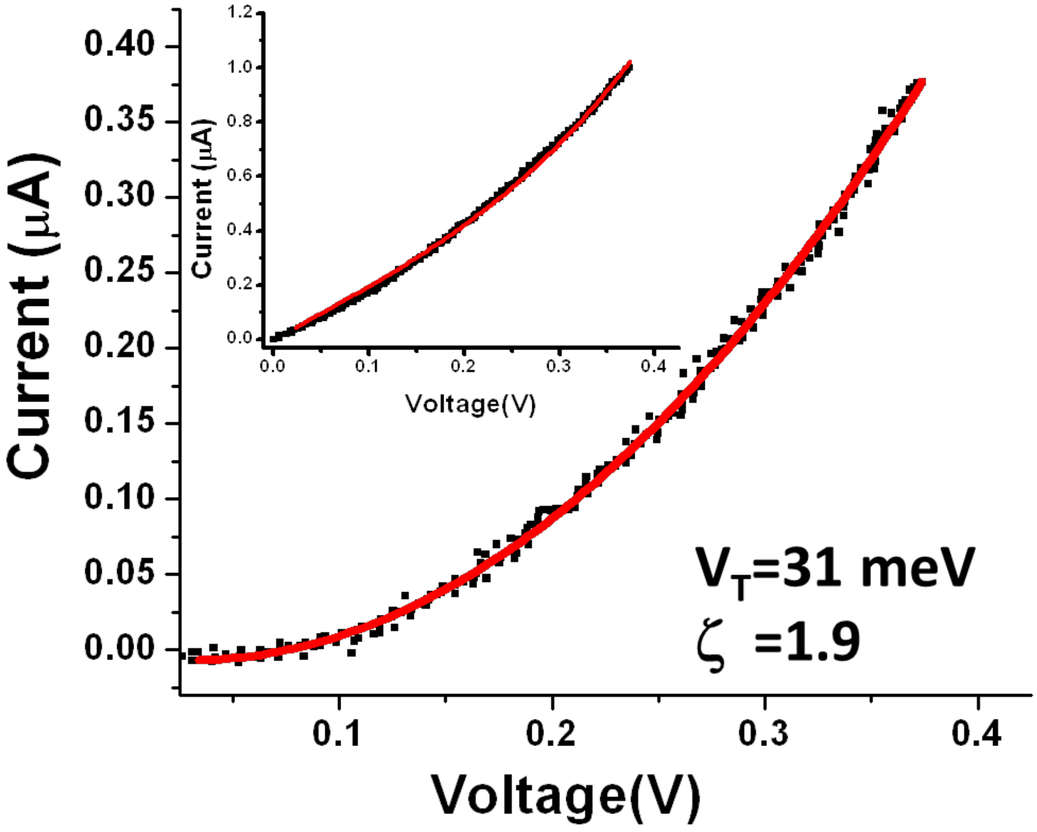

Figure S4. Likharev model fit for current (μA) vs voltage (V) at 80 K for GQD-Bacterial spore device. The threshold Coulomb blockade voltage (V_T) is 31 meV, and geometric factor (ζ)~1.9/ The inset shows the raw data with background conductivity.

V. Thermal barrier analysis of Graphene cytobot:

The activation barrier of GQD-Bacterial spore device is evaluated using the Arrhenius equation model $\frac{I}{V} \propto exp(-\frac{E_a}{k_B T})$, where I is measured current (A), V is applied bias voltage (V), k_B is the Boltzmann constant, T is the absolute temperature, and E_a is thermal activation barrier.

$$\frac{I}{V} \propto exp(-\frac{E_a}{k_B T})$$

Linear relationship equation:

$$\ln\left(\frac{I}{V}\right) \propto \left(-\frac{E_a}{k_B}\right)\left(\frac{1}{T}\right)$$

where E_a = 35 meV is obtained from the slope of logarithmic plot of $\left(\frac{I}{V}\right)$ as a function of $\left(\frac{1}{T}\right)$.

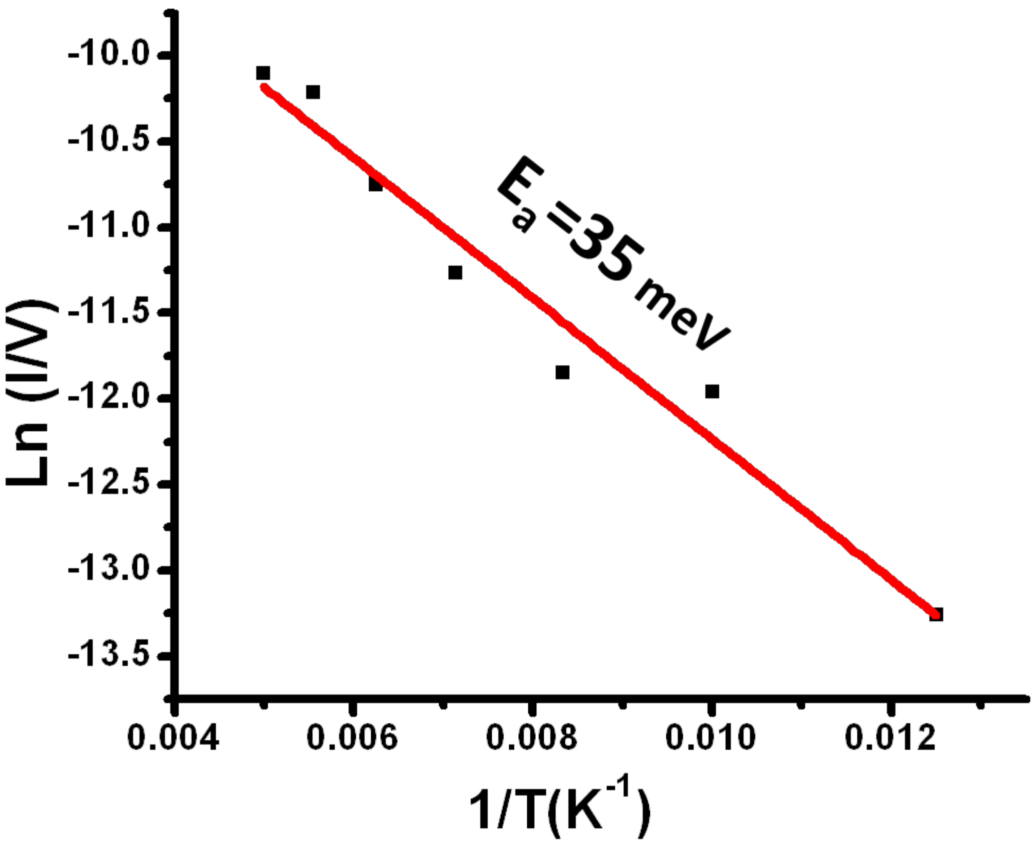

Figure S5. shows the temperature responds to $\left(\frac{I}{V}\right)$ for GQD-Bacterial spore device. From the slope, the activation energy (E_a = 35 meV) is found, which is which is close to blockade threshold voltage V_T = 31 meV.

VI. Capacitance Measurement:

The impedance measurements were carried out by Agilent LCR system (Model 4287A).

The GQD-Spore device is prepared as described in the manual script. The inter GQD capacitance (C_B) and sinusoidal angular frequency (ω) are measured through LCR meter. The 4 wire measurement set up is shown in the schematic.

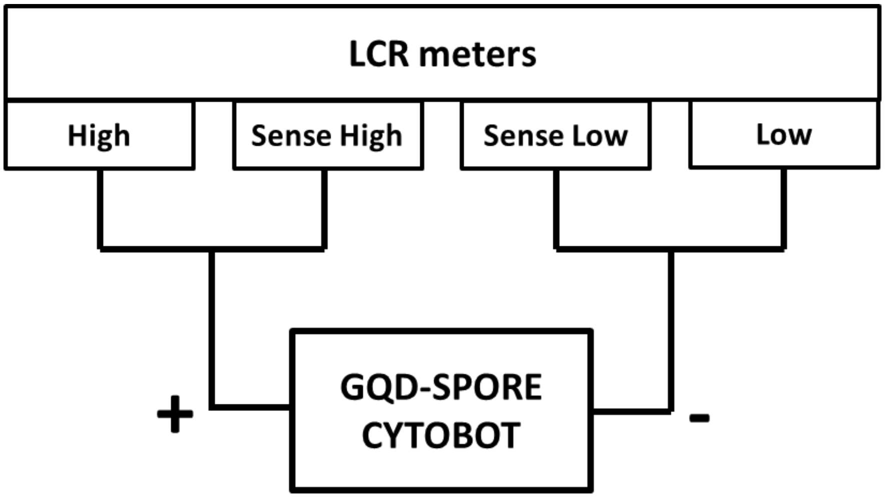

Figure shows the 4 wire measurement of the inter GQD capacitance (C_B) and sinusoidal angular frequency (ω). The biaxial cables are used in the connection to minimize the contact resistance, and inherent electrical noise.

The equivalence impedance of the GQD-Spore device can be expressed as:

$$\frac{1}{Z} = (R)^{-1} + \left(\frac{1}{i\omega C_B}\right)^{-1}$$

$$Z = \left(\frac{1}{\frac{1}{R} - i\omega C_B}\right)$$

$$Z = \left(\frac{1 - R\omega C_B i}{\omega^2 {C_B}^2 + \frac{1}{R^2}}\right)$$

$tan(\theta) = -\omega C_B \mathrm{R}$

We can express the above linear equation as:

$y = ax$ ($y = -tan(\theta)$; $x = \omega$); $slope = a = RC_B$ and $intercept =$ 0. The impedance of the spore without the GQDs was first measured followed by deposition of GQDs and re-measurement of the impedance versus frequency for the device. The two impedances were subtracted (via parallel impedance equation) to determine Z and tan(θ) at different frequencies.

1. The phase angle versus frequency relationship for the resisto-capacitive GQD tunneling junction (R, C_S) is: $-tan(\theta) = (RC_B)\omega$. This provides the slope or RC_B.

2. From the slope (with intercept = 0), the capacitance between GQDs was calculated to increase from 8.54 to 9.61 pF for atmospheric humidity to high vacuum (~3 Torr). The resistance was measured at 0.1 Hz frequency: 13.81 KΩ and 12.24 KΩ at atmospheric pressure and under vacuum, respectively.

3. Capacitance increase = 1.12 folds

4. Change in tunneling distance was estimated from Mass-Trabsfer model equation

$$= \frac{a_{Atm}}{a_{Vac}} = \frac{\sqrt[3]{1+fP_{atm}+f'(0){P^2}_{atm}}}{\sqrt[3]{1+fP_{vac}+f'(0){P^2}_{vac}}} = 4.8$$

5. The ratio between the dielectric constant of the tunnel junction $= (\frac{\varepsilon_{atm}}{\varepsilon_{vac}} = (\varepsilon_w/\varepsilon_{NAM})f + (1-f))$. Here, ε_w and ε_{NAM} are the dielectric constants of water (80.4) and dry NAM-junction (assumed 3), and *f* is the fraction of water coverage in the tunnel junction (parallel junctions).

6. Then we found the value of *f* which will provide the same change in tunneling distance as estimated from point (4) or $\frac{C_{atm}}{C_{vac}} = \frac{\varepsilon_{atm}}{\varepsilon_{vac}}\frac{a_{Vac}}{a_{Atm}}$. *f* = 27.5% (consistent with about 50% hydrophilic sites on spore-wall and the 45% humidity (rH) (~ 22.5% at equilibrium)).

7. For the capacitance, there are two competing forces: reduction in tunneling distance increasing the capacitance and the reduction in water adsorbed reducing the capacitance.

VII. Response Time:

The response of a polymer to swelling is directly related to interaction potential between the polymer's molecular groups and water (J. Chem. Phys. 1943, 11, 521.; J. Appl. Phys. 107, 103535 2010). While the flux of solvent is dependent on the time dependent chemical potential (μ) as $J = -\frac{CD}{kT}\left(\frac{dR}{dr}\right)^2 \frac{d\mu}{dR}$; the equilibrium force equation provides: $\frac{d\left(\frac{r\sigma_r}{R}\right)}{dR} + \frac{\frac{r\sigma_r}{R} - \frac{dr}{dR}\sigma_\theta}{R} = 0$, where σ_r and σ_θ are the pressure terms in the radial and angular directions. Our previous experiments with regular polymers show very slow response (see below). We speculate that this is a result of combined high chemical-potential of spore-constituents and the fast diffusion through its membrane (hydrophobic/hydrophilic and selective transport of water). For poly-allyl-hydrochloride fiber with GQDs, the response is > 30 seconds, while for spore the response is a few seconds (~3 s). These are approximately estimated to be equal to the time it take for the device to reach steady state conductivity.

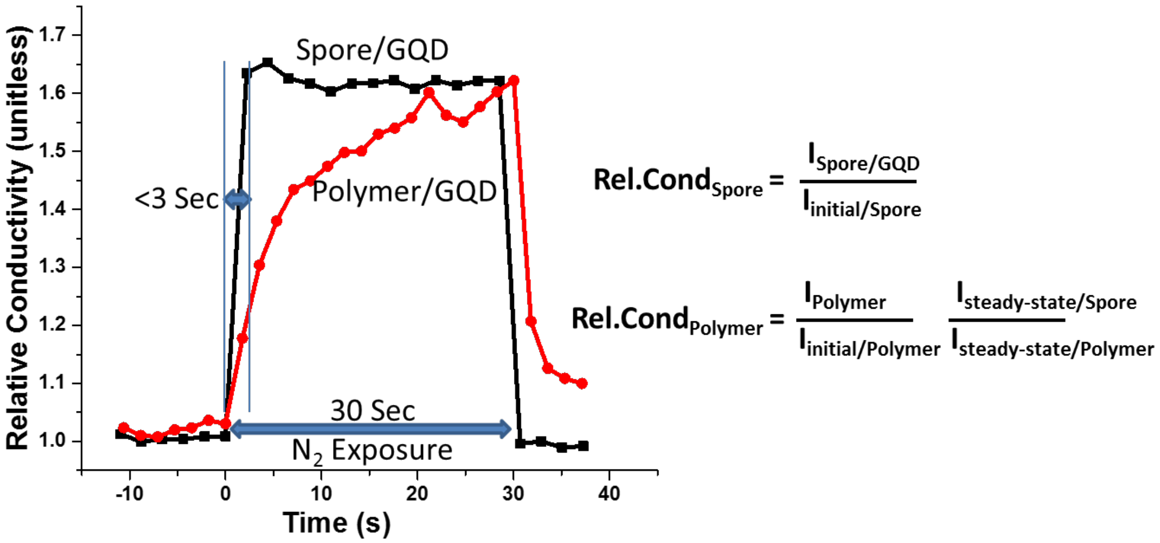

Figure S6: Response comparison between spore/GQD and polymer/GQD (Nano Letter, 13 (4), 1757–1763, 2013).

Further, the sharp actuation (with low phase-difference) implies a higher sensitivity. Based on the time scale to saturation, we estimate an improvement of >10 folds in response time.

VIII. More Devices:

(A) Device response with increased time of deposition of EfGQDs (24 h).

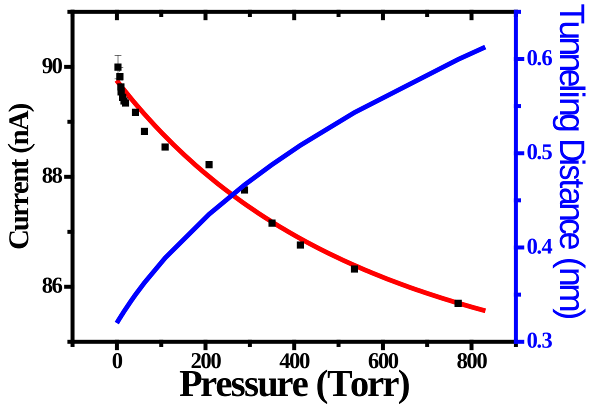

Figure: Current versus pressure with mass-transfer model fit; and tunneling distance versus pressure fit for device fabricated with increase time of deposition of EfGQDs.

(B) Device response with reduced time of deposition of EfGQDs (6h)

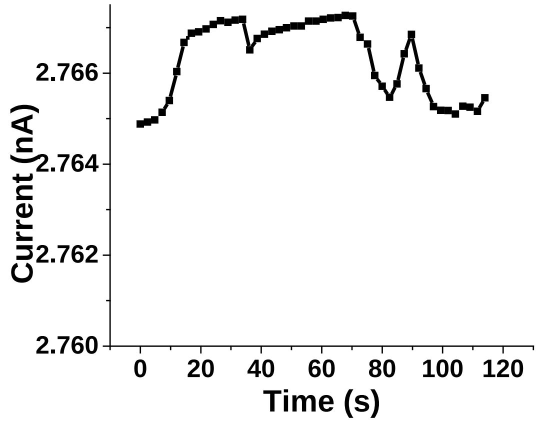

Figure: Lower deposition time: Change in conductivity of the device *via* exposure to nitrogen atmosphere (0 to 70 seconds) at 0.1 V. The device was found to respond weakly and exhibited noise (notice the dip at ~31 s and the rise at ~90s).

Analysis of increased deposition time: (a) The increase in deposition time leads to reduced tunneling distance at 0 pressure; (b) The total conductivity at 0 pressure increases due to reduced tunneling distance; and (c) The change in conductivity (response) reduced, attributed to less initial tunneling distance (at 1 atm pressure); and increase in energy required to reduce the tunneling gap below 0.4 nm.

Analysis of reduced deposition time: With reduced deposition, (a) the current levels become very small, attributed to reduced number of channels and higher tunneling distances. (b) The increase in conductivity

in dry condition is reduced to 2 pA (which is in the noise range for our system); and (c) we also observed that the device becomes noisy, probably due to the migration of GQDs.
